# Relationships between prolonged physical and social isolation during the COVID-19 pandemic, reduced physical activity and disability in activities of daily living among people with advanced respiratory disease

**DOI:** 10.1177/14799731211035822

**Published:** 2021-08-12

**Authors:** Lucy Fettes, Joanne Bayly, Leonora Michelle de Bruin, Malini Patel, Stephen Ashford, Irene J Higginson, Matthew Maddocks

**Affiliations:** 1 Cicely Saunders Institute of Palliative Care, Policy and Rehabilitation, 4616 King’s College London, London, UK; 2 Regional Hyper-acute Rehabilitation Unit, Northwick Park Hospital, London North West University Healthcare NHS Trust, Harrow, London, UK; 3 Centre for Nursing Midwifery and Allied health Research and the National Hospital for Neurology and Neurosurgery, 8964 University College London Hospitals, Holborn, London, UK

**Keywords:** Activities of daily living, COVID-19, disability, rehabilitation, respiratory disease, social isolation

## Abstract

In people with advanced respiratory disease, we examined (i) the impact of COVID-19–related physical and social isolation on physical activity and (ii) relationships between time spent in isolation and disability in activities of daily living. Cross-sectional analysis was conducted in adults with advanced non-small cell lung cancer, chronic obstructive lung disease or interstitial lung disease. Measures included change in physical activity since physically and socially isolating (Likert scale) and disability (Barthel Index and Lawton–Brody IADL scale) or difficulty (World Health Organisation Disability Assessment Schedule-2.0) in daily activities. Multiple logistic regression was used to examine factors associated with disability in daily activities. 194/201 participants were isolating for a median [IQR] 5 [3–8]-month period, often leading to lower levels of physical activity at home (*n* = 94, 47%), and outside home (*n* = 129, 65%). 104 (52%) and 142 (71%) were not fully independent in basic and instrumental activities of daily living, respectively. 96% reported some degree of difficulty in undertaking daily activities. Prolonged physical and social isolation related to increased disability in basic (r = −0.28, *p* < 0.001) and instrumental (r = −0.24, *p* < 0.001) activities of daily living, and greater difficulty in daily activities (r = 0.22, *p* = 0.002). Each month spent in physical or social isolation was independently related to disability in basic activities of daily living (odds ratio [OR], 1.17 [95% CI: 1.03–1.33], *p* = 0.013). These findings suggest disability in daily activities is associated with prolonged physical or social isolation, which may present as difficulty in people who are fully independent. Post-isolation recovery and rehabilitation needs should be considered for all people deemed extremely clinically vulnerable.

## Introduction

Coronavirus (COVID-19) was declared a global pandemic by the World Health Organization on 11th March 2020.^1^ About one in five individuals worldwide are considered at increased risk of severe COVID-19 infection due to underlying health conditions including respiratory disease, encouraging countries to put policies in place to protect those at increased risk.^2^ In the United Kingdom, as part of government policy, individuals fulfilling these high-risk criteria were classed as ‘extremely clinically vulnerable’ and physical and social isolation (shielding) was advised.^3^ This included many of the estimated 85,000 people living with lung cancer, 1.2 million people living with chronic obstructive pulmonary disease (COPD) and 32,500 people living with interstitial lung disease (ILD).^4^ The Global Burden of Disease Study reports non-malignant and malignant respiratory disease to be the third and fourth leading cause of death and productive life lost due to disability in the United Kingdom in 2019, respectively, which is higher than any other country with similar health system performance.^5^ Therefore, protecting this population from the severe risk of COVID-19 and preventing disability is a particular concern in the United Kingdom.

Physical and social isolation refers to a lack of contact with society^6^ and has been found to decrease physical activity and increase sedentary behaviour.^7^ Physical and social isolation adversely affects psychosocial and mental health functioning^8^ and results in functional impairments^6^ and deconditioning.^9^ In people with advanced respiratory disease, it is currently unclear how prolonged physical and social isolation may impact disability, and health- and social-care services post-pandemic, whether or not they contract the virus.^10^

Furthermore, COVID-19 guidance has caused disruption to treatment or disease management delivery, including reduced access to cancer therapies and rehabilitation.^11^ On the other hand, there has been a significant reduction in exacerbations and improvement in symptoms in COPD patients, possibly relating to less exposure to respiratory viruses, and/or a strict adherence to physical and social isolation.^12^ However, there was also a reluctance to seek medical attention during the pandemic by individuals considering themselves clinically vulnerable.^13^

The World Health Organization (WHO) defines disability as ‘any condition of the body or mind (impairment) that makes it more difficult for the person with the condition to do certain activities (activity limitation) and interact with the world around them (participation restrictions)’.^14^ This is characterised by a complex relationship between an individual’s health condition, the environment in which they live and personal attributes.^14^ Activities of daily living (ADLs) describe a collection of skills required to live independently.^15^ Activities of daily livings can be classified as basic (e.g. feeding, dressing and continence) or instrumental (e.g. shopping, housework and transportation).^15^ Activities of daily living disability can be considered in terms of ADL dependency; a reliance on others, or ADL difficulty, which describes an increased difficulty to manage ADLs independently. Both have been linked to poorer clinical outcomes and quality of life.^16^

This study aimed to (i) describe the impact of physical and social isolation on an individual’s level of physical activity; (ii) examine the relationship between time spent in physical and social isolation, disability in basic and instrumental ADLs and difficulty managing daily activities; and (iii) examine factors associated with disability in ADLs in people with advanced respiratory disease during the COVID-19 pandemic.

## Methods

### Study design

We report baseline data of a prospective cohort study, following the STROBE guidelines.^17^ The study was registered on the ISRCTN registry (ISRCTN14159936), and ethical approval was granted by the London Camberwell St Giles Research Ethics Committee (ref 19/LO/1950).

### Recruitment setting

We recruited from 12 sites across England from July 2020 to January 2021, including eight acute NHS trusts, three hospices and the British Lung Foundation. Recruitment settings included hospital medical, respiratory or oncology wards; outpatient lung cancer or respiratory clinics; and hospice/palliative care inpatient, outpatient and community services. The study was advertised through the British Lung Foundation members’ forum.

### Eligibility criteria

Inclusion criteria were adults with a diagnosis of either (i) inoperable stage III or IV non-small cell lung cancer; (ii) severe or very severe COPD, defined by FEV_1_ < 50% predicted^18^; or (iii) advanced ILD, defined by carbon monoxide transfer factor (TLCO/DLCO) level of < 40% or FVC < 50% predicted.^19^ Patients were excluded if they lacked capacity to consent, were unable to complete the survey in English or had a clinician-estimated life expectancy of less than 1 month.

### Recruitment strategy

Eligible patients were identified from their medical notes and approached by a member of their clinical team at a routine face-to-face or telephone consultation. Verbal consent was taken for the research team to contact them about the study. Alternatively, members of the British Lung Foundation could self-refer directly to the researcher. Study information was posted to the participant and followed a week later by a telephone call to take informed verbal consent and complete the baseline questionnaire if they agreed to participate.

### Variables and measures

Demographic data and participant characteristics were collected, including diagnosis, age, gender, ethnicity, education level, living status and location, carer support, Charlson Co-morbidity Index score,^20^ Australian Karnofsky Performance Status^21^, and symptom severity (Palliative Outcomes Scale-symptoms),^22^ along with the following patient-reported variables of interest.

#### Time spent in physical and social isolation (in months):

This was collected by asking participants whether they are, or/and have been physically or socially isolating and how long for, including dates of isolation period based on dated government letters.

#### Change in physical activity since physically or socially isolating:

This was *measured* using a 5-point Likert scale: a lot less, a little less, no change, a little more or a lot more in (i) physical activity inside the home and (ii) physical activity outside the home. The Likert scale is one of the most fundamental and frequently used psychometric tools for scaling responses in survey research where response to change is common.^23,24^

#### Disability in carrying out basic ADLs:

This was measured using the Barthel Index, consisting of 10 items (bowel incontinence, toilet use, grooming, feeding, mobility, bladder incontinence, dressing, bathing, stairs, and transfers).^25^ Domains are scored according to the level of physical assistance required to perform the daily task with individual scores varying between 0–1, 0–2 and 0–3, depending on the number of options per item. A combined total score of all 10 items ranges from 0 to 20. A score of zero corresponds to full ADL dependence, whilst 20 reflects full independence.^25^ A change of 1.85 in stroke and 3.6 in older people indicates a minimal clinically important difference (MCID) in patient reported Barthel Index score.^26^

#### Disability in carrying out instrumental ADLs:

This was measured using the Lawton–Brody IADL scale, an 8-item categorical measure (ability to use the telephone, shopping, food preparation, housekeeping, laundry, mode of transportation, responsibility for own medication and ability to manage finances).^27^ Each item has a range of three to five responses ranging from fully independent to fully dependent. Each response is scored one if independent or 0 for anything other than independent. A summary score ranges from 0 (low function, dependent) to 8 (high function, independent); a lower score indicates greater disability.^27^ The MCID for the Lawton–Brody IADL scale lies around half a point.^28^

#### Difficulty in managing daily activities:

This was measured using the World Health Organisation Disability Assessment Schedule (WHODAS-2.0).^29^ The WHODAS-2.0 measures disability in terms of difficulty managing ADLs independently, as opposed to the Barthel Index and Lawton–Brody IADL scale which measure disability in terms of dependency on others. This index consists of six domains (cognition, mobility, self-care, getting along with people, life activities and societal participation). Life activities consist of two sections: household activities and work activities; the latter is optional to include and was therefore excluded from this analysis. All items are scored on a scale of activity difficulty ranging from 1 to 5: none [1], mild [2], moderate [3], severe [4] and extreme or cannot do [5]. The cognition domain is made up of six items; mobility and getting along with people, each have five items; self-care and household activities, each have four items; and societal participation has seven items. Domain scores were totalled to produce a WHODAS summary score, where 32 reflects no difficulty and 160 extremely difficult (excluding the work domain).^29^ A WHODAS summary score of 32 = no difficulty, 33–64 = mild difficulty, 65–96 = moderate difficulty, 97–128 = severe difficulty and 129–160 = extreme difficulty or cannot do.^29^ The WHODAS-2.0 is the current leading measure of disability worldwide; however, a MCID for the WHODAS-2.0 has not yet been established.^30^

### Sample size

A sample size of 200 is sufficient to achieve a precision of at least 8% in the estimation of prevalence of ADL disability, based on assumed prevalence to be around 50%.^31,32^ This sample size would also be sufficient to detect a significant correlation of ≥ 0.20.^33^

### Data analysis

Participant characteristics and change in physical activity during physical and social isolation were summarised using descriptive statistics. Diagnosis was split into two groups: malignant (lung cancer) or non-malignant (COPD or ILD). Participants with both a malignant and non-malignant diagnosis were classified in the malignant group. The Mann–Whitney U-test was used to compare the two diagnostic groups and differences between those who did and did not receive a government (GOV) letter of request to physically and socially isolate.

Univariate associations between (i) months spent physically and socially isolating, (ii) Barthel Index total score, (iii) Lawton–Brody IADL Scale total score and (iv) WHODAS-2.0 summary score were calculated using the Spearman’s rho test. Disability in basic ADLs and instrumental ADLs were each split into two groups: (i) fully independent (Barthel Index = 20/Lawton–Brody = 8) and (ii) disability (Barthel Index < 20/Lawton–Brody < 8). Difficulty in managing ADLs measured by the WHODAS summary score was defined by level of disability (fully independent/disabled) in basic and instrumental ADLs separately.

Our primary dependent variable in logistic regression analysis was (a) whether the participant had disability in basic ADLs (Barthel Index < 20) or was fully independent (Barthel Index = 20) and (b) whether the participant had disability in instrumental ADLs (Lawton–Brody IADL Scale < 8) or was fully independent (Lawton Brody IADL Scale = 8). Explanatory variables considered for the model were based on a recent systematic review^34^ and included diagnosis, time spent physically and socially isolating, age, gender, living status and symptom severity. The model included complete cases only.

## Results

**Figure 1.**
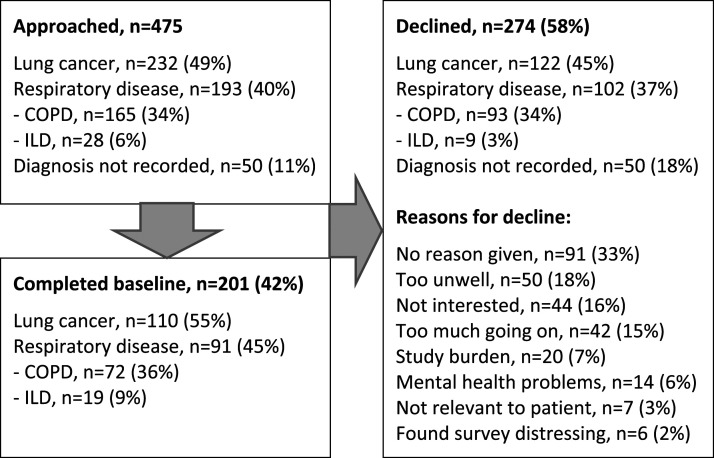
Study flow diagram.

201 participants were recruited, 110 (55%) with malignant respiratory disease and 91 (45%) with non-malignant (72 (36%) COPD and 19 (9%) ILD), respectively. The study flow and participant characteristics are presented in Figure 1 and Table 1. Data were missing on physical and social isolation and disability in daily activities (WHODAS-2.0) for one participant each. For all participants, the median [IQR] disability in independence in basic ADLs, instrumental ADLs and difficulty in daily activities was 19 [17–20], 7 [3–10] and 57 [46–79], respectively, illustrating overall mild disability (Table 1).

**Table 1.** Participant characteristics.

	All diagnoses *n* = 201	Malignant respiratory disease, *n* = 110 (55%)	Non-malignant respiratory disease, *n* = 91 (45%)	Difference between groups (*p* value)
Age	69 [63–75]	68 [61–72]	72 [66–77]	< 0.001
Female	91 (45%)	51 (46%)	40 (44%)	0.73
White British	191 (95%)	105 (95%)	86 (95%)	0.76
Education above secondary school	93 (46%)	52 (48%)	41 (45%)	0.85
Lives alone	68 (34%)	36 (33%)	32 (35%)	0.72
Inpatient/residential care	4 (2%)	0	4 (2%)	0.03
Formal caregiver	29 (14%)	11 (10%)	18 (20%)	0.05
Informal caregiver	112 (56%)	54 (50%)	58 (64%)	0.05
Physiotherapy input within the last month	20 (10%)	6 (5%)	14 (16%)	0.02
Occupational therapy input within the last month	10 (5%)	3 (3%)	7 (8%)	0.10
Charlson Co-morbidity Index score	7 [3–10]	9 [7–13]	3 [2–5]	< 0.001
Australian Karnofsky Performance Status	70 [60–80]	80 [60–90]	60 [60–70]	< 0.001
Received GOV letter to physically and socially isolate	174 (87%)	91 (84%)	83 (91%)	0.14
Currently physically and socially isolating	143 (71%)	72 (65%)	71 (78%)	0.05
Have spent time in physical and social isolation	194 (97%)	104 (95%)	90 (99%)	0.15
Months spent in physical and social isolation	5 [3–8]	4 [3–6]	6.5 [4–9]	< 0.001
Total Barthel Index score (basic ADLs)	19 [17–20]	20 [19–20]	18 [15–19]	< 0.001
Lawton–Brody IADL score (instrumental ADLs)	7 [5–8]	7 [6–8]	5 [4–7]	< 0.001
WHODAS summary score	57 [46–79]	49 [40–62]	73 [57–87]	< 0.001
*Cognition*	7 [6–10]	6 [6–8]	8 [6–12]	< 0.001
*Mobility*	13 [7–17]	9 [6–13]	17 [13–19]	< 0.001
*Self-Care*	5 [4–9]	4 [4–5]	6 [5–11]	< 0.001
*Getting along with people*	9 [4–13]	7 [5–9]	10 [8–13]	< 0.001
*Household activities*	9 [4–13]	6 [4–10]	12 [9–18]	< 0.001
*Societal participation*	17 [12–21]	15 [11–20]	19 [14–22]	< 0.001
Symptom severity (Palliative Outcomes Scale-symptoms)	10 [5.5–15]	7 [4–13]	11.6 [8–18]	< 0.001

ADLs: Activities of daily livings; WHODAS: World Health Organisation Disability Assessment Schedule; GOV: Government.

Values are *n* (%) or [median, IQR]; Missing data: physical and social isolation, *n* = 1, WHODAS-2.0, *n* = 1.

Participants with non-malignant respiratory disease had significantly greater dependency in basic ADLs, instrumental ADLs and increased difficulty in daily living (all *p* < 0.001), compared with participants with malignant respiratory disease. They were also significantly older, had a lower functional performance status and higher symptom severity.

During the first wave of the COVID-19 pandemic, 174 (87%) participants received a letter of request from the government to physically and socially isolate, which was not significantly different between those with malignant or non-malignant respiratory disease (*p* = 0.14). Differences between participants who did and did not receive this letter are presented in Supplementary Table 1. We found those who received the letter were more symptomatic (*p* = 0.003), more likely to physically and socially isolate (*p* < 0.001) and reduce their participation in society (*p* = 0.002) than those who did not receive the letter.

**Figure 2.**
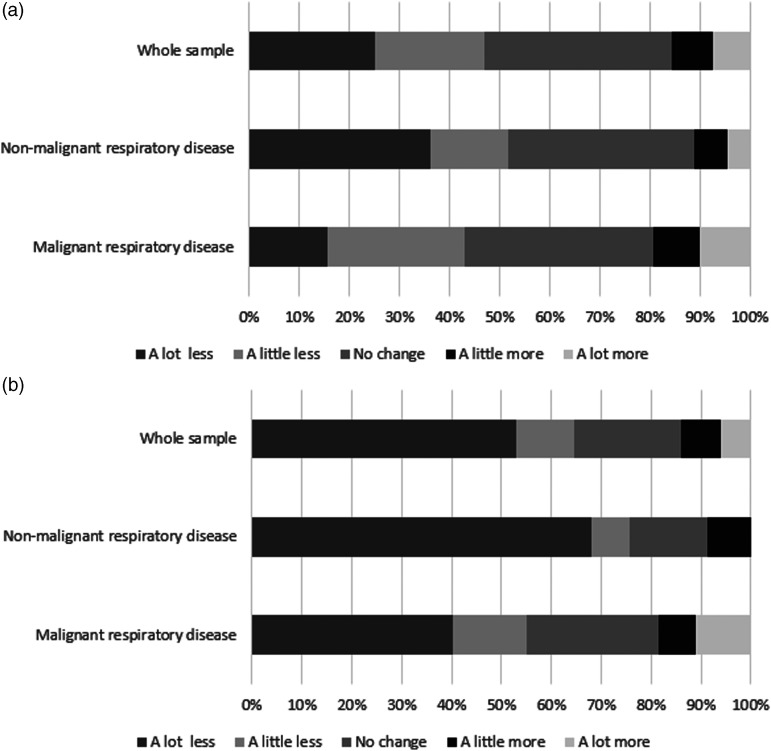
Change in physical activity during physical and social isolation. (a) Change in physical activity inside the home; (b) Change in physical activity outside the home

Almost all participants (194/97%) had spent time physically and social isolating for a median [IQR] period of 5 [3–8] months at the time of assessment. During physical and social isolation, 94 (47%) participants were less physically active at home (Figure 2(a)). Physical activity outside the home was lower in 129 (65%) participants (Figure 2(b)). Patients with non-malignant respiratory disease were significantly less physically active than patients with malignant respiratory disease, inside (*p* = 0.02) and outside (*p* = 0.004) the home.

**Figure 3.**
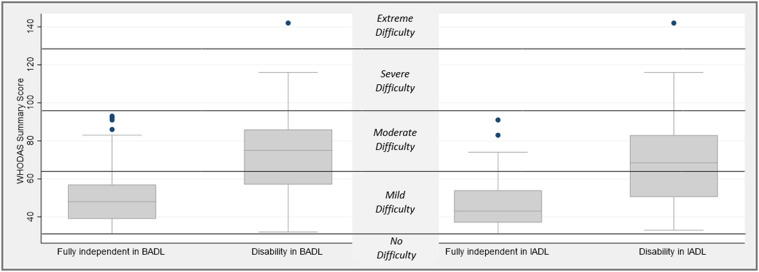
Difficulty in daily activities (WHODAS summary score (median [IQR])) in patients with advanced respiratory disease who have full independence or disability in basic (BADL) and instrumental (IADL) activities of daily living. WHODAS: World Health Organisation Disability Assessment Schedule.

97 (48%) participants were fully independent in basic ADLs, and 59 (29%) were fully independent in instrumental ADLs. 197 (96%) participants had difficulty managing daily activities (median [IQR]) including those fully independent in basic ADLs (48 [39–57]) or instrumental ADLs (43 [37–54]) (Figure 3). Only 10% and 5% of participants received physiotherapy or occupational therapy interventions, respectively, within the last month.

**Figure 4.**
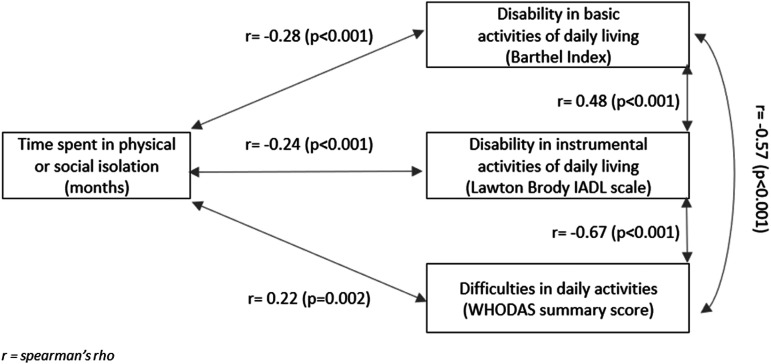
Univariate associations between time spent in physical or social isolation, disability in basic activities of daily living, disability in instrumental ctivities of daily living, and difficulties in daily activities.

A longer time in physical or social isolation was weakly associated with increased disability (lower Barthel Index or Lawton–Brody total score) in basic (r = −0.28, *p* < 0.001) and instrumental ADLs (r = −0.24, *p* < 0.001), and greater difficulty (higher WHODAS summary score) in daily activities (r = 0.22, *p* = 0.002) (Figure 4). Moderate relationships were found between less independence in basic ADLs, less independence in instrumental ADLs and greater difficulty in daily activities.

The multivariable analysis (Table 2) showed that disability in basic ADLs was related to prolonged physical and social isolation (odds ratio [OR], 1.17 [95% CI: 1.03–1.33], *p* = 0.01), non-malignant respiratory disease (odds ratio [OR], 4.00 [95% CI: 1.20–8.14], *p* < 0.001) and increased symptom severity (odds ratio [OR], 1.12 [95% CI: 1.06–1.19], *p* < 0.001). Disability in instrumental ADLs was related to non-malignant respiratory disease (odds ratio [OR], 3.6 [95% CI: 1.41–7.10], *p* = 0.005) and increased symptom severity (odds ratio [OR], 1.14 [95% CI: 1.07–1.22], *p* < 0.001). Both models were adjusted for months spent in physical and social isolation, diagnosis, age, gender, living status and symptom severity.

**Table 2.** Adjusted associations with disability in activities of daily living using multivariable logistic regression.

a) Disability in basic activities of daily living (*n* = 199)	Odds ratio [OR]	[95% conf. Interval]		*p* value
Months spent in physical and social isolation	1.17	1.03	1.33	0.01
Non-malignant respiratory disease (COPD or ILD)	4.00	1.20	8.14	< 0.001
Symptom severity (Palliative Outcomes Scale-symptoms)	1.12	1.06	1.19	< 0.001
Age	1.02	0.98	1.06	0.32
Female	1.48	0.74	2.96	0.26
Live alone	1.70	0.82	3.52	0.15
_Cons	0.01	0.0006	0.25	0.004
b) Disability in instrumental activities of daily living (*n* = 200)	Odds ratio [OR]	[95% conf. Interval]		*p* value
Months spent in physical and social isolation	1.19	0.59	2.41	0.63
Non-malignant respiratory disease (COPD or ILD)	3.16	1.41	7.10	0.005
Symptom severity (Palliative Outcomes Scale-symptoms)	1.14	1.07	1.22	< 0.001
Age	1.03	0.99	1.07	0.21
Female	1.19	0.59	2.41	0.63
Live alone	0.68	0.33	1.41	0.30
_Cons	0.08	0.004	1.35	0.08

COPD: chronic obstructive pulmonary disease; ILD: interstitial lung disease.

Reference group (a) is disability in basic activities of daily living (Barthel Index < 20); Reference group (b) is disability in instrumental activities of daily living (Lawton–Brody IADL Scale < 8); All variables in this table have been dichotomised, except months spent in physical and social isolation, symptom burden and age, which were treated as continuous variables.

## Discussion

### Main findings

In our cross-sectional analysis of 201 participants with advanced respiratory disease, physical and social isolation was highly prevalent. We report several main findings. Firstly, physical and social isolation has resulted in lower levels of physical activity. Secondly, disability in activities of daily living is common in advanced respiratory disease and even those who are fully independent in ADLs have difficulty managing daily activities independently. Finally, disability in basic activities of daily living independently relates to increased time spent in physical or social isolation, and both basic and instrumental activities of daily living independently relate to non-malignant respiratory disease and increased symptom severity*.*

### Contributions to the literature

Our findings contribute to the literature in several ways. Firstly, we identified that nearly all participants with advanced respiratory disease spent time in physical and social isolation due to the pandemic, resulting in a reduction in their usual physical activity. This corroborates a small cohort study of 10 COPD patients who had a significant reduction in their level of physical activity during the first 3 months of the pandemic while under instructions to physically and socially isolate following a course of pulmonary rehabilitation.^35^ Furthermore, we found the impact on reduced activity in non-malignant respiratory disease was significantly greater than malignant respiratory disease. However, even pre-pandemic, over time, physical activity in COPD has been shown to follow a downwards trajectory and exacerbated by sedentary behaviour.^36^ In older patients with advanced cancer, perceptions of physical activity are positive, and periods of reduced activity usually occur during cancer treatment.^37^

Secondly, we identified people who may be indirectly affected by the pandemic. People who spend longer in physical and social isolation experience greater disability in basic ADLs. Also, those with disability in basic and instrumental ADLs have a higher symptom severity and/or a non-malignant respiratory diagnosis. This may arise from feelings of vulnerability from COVID-19^13^ where reduced confidence to participate in normal daily activities leads to deconditioning and functional impairment.^6,9^ Symptoms restricting disability are common in advanced disease,^38^ and higher symptom severity is associated with a housebound status, significantly limiting a persons’ ability to carry out activities involving socialising and participating in the community.^39^ This highlights the contribution of health, environmental and personal factors in the development of disability.^14^

Thirdly, we found that despite some participants being fully independent in activities of daily living they often experienced ‘difficulty’ in managing their daily activities independently. Participants in our study may be struggling independently due to lack of or reluctance to accept help due to restrictions on social contact, particularly if living alone. This may be missed by only measuring dependency. It is also plausible that difficulty pre-empts disability, therefore recognising and addressing difficulty in daily activities may help to maintain independence and prevent dependency. Helping people to continue to live independently at home as their condition progresses could potentially reduce or delay the need for social care. This is supported by the Health and Retirement Study that identified nursing home placements could be strongly predicted by difficulty bathing.^40^

### Clinical implications

It is important to recognise the effect limited access to rehabilitation may have had on disability in daily activities in advanced respiratory disease. During the pandemic, rehabilitation is reported to have been the most disrupted health service, often being deemed non-essential.^11^ This is reflected in our findings where less than a fifth of participants received physiotherapy or occupational therapy interventions despite most participants reporting difficulty in managing daily activities independently. Online delivery has been found to be acceptable during this time,^41,42^ but there are access challenges for patients who have limited knowledge or availability to these resources.^43^

In addition, social support provision is likely to have been impacted by COVID-19 guidelines. This included difficulty getting the necessary basics such as food, difficulty accessing healthcare services for support and feelings of loneliness.^44^ Social support can be considered a protective psychological factor against a decline in mental and physical health–related quality of life.^45^ Two cohort studies have identified that poorer satisfaction with social support is associated with greater difficulties in instrumental activities of daily living in people with chronic conditions, where the quality of social support was identified to be of greater importance than the quantity.^46^ Among COPD patients, low support levels have been associated with depression and physical symptom deterioration.^47^ Positively, physical and social isolation may reduce hospitalisation due to reduction in exacerbations in COPD patients.^12^ However, patients with cancer may have suffered delays in treatment and less access to support due to restrictions on visitors, which may accelerate decline.^48^

Consequently, physical and social isolation and reduced rehabilitation threatens a post–COVID-19 wave of disability in people with advanced respiratory disease. Addressing disability is important as it is known to lead to increased hospital stay and discharge to a care facility,^49^ putting increased strain on already stretched health- and social-care services. Moving forward, health- and social-care services need to consider post–COVID-19 recovery and rehabilitation for all people deemed extremely clinically vulnerable.^50^ To help identify need, we recommend consideration is given to the following individual risk factors: (i) length of time spent in physical and social isolation, (ii) presenting difficulty and not only disability in daily activities, (iii) symptom severity and (iv) level of social support, with a heightened awareness in non-malignant respiratory disease. Further, we propose strategies are considered to (i) minimise time spent in isolation, (ii) maintain physical activity, (iii) continue rehabilitation services or/and offer online alternatives, and (iv) increase social support. More research is required to ensure their success.

### Study strengths and limitations

We recruited a large sample of patients with advanced respiratory disease across multiple sites to increase generalisability of the findings. We report baseline data only, identifying associations and not causative relationships. Potential bias includes varying time of individual data collection, fluctuating COVID-19 guidelines over the recruitment time period, use of subjective measures over objective measurement and response or recall from self-reported measures. In addition, instrumental ADLs were compromised by the context of COVID-19 lockdown restrictions themselves and therefore this regression analysis should be interpreted with caution. Analysis of the longitudinal data from the ongoing cohort study will add a valuable understanding of the impact of physical and social isolation on disability over time.

## Conclusion

Evidence from this study suggests that disability is associated with prolonged physical or social isolation. This implies this population with advanced respiratory disease is deconditioning as an indirect result of the pandemic. Consideration needs to be given to post–COVID-19 recovery and rehabilitation for all people deemed extremely clinically vulnerable. Strategies to better handle the rehabilitation needs of those in physical and social isolation in light of future pandemics need to be prepared.

## Supplemental Material

sj-pdf-1-crd-10.1177_14799731211035822 – Supplemental Material for Relationships between prolonged physical and social isolation during the COVID-19 pandemic, reduced physical activity and disability in activities of daily living among people with advanced respiratory disease Click here for additional data file. Supplemental Material, sj-pdf-1-crd-10.1177_14799731211035822 for Relationships between prolonged physical and social isolation during the COVID-19 pandemic, reduced physical activity and disability in activities of daily living among people with advanced respiratory disease by Lucy Fettes, Joanne Bayly, Leonora Michelle de Bruin, Malini Patel, Stephen Ashford, Irene J Higginson and Matthew Maddocks in Chronic Respiratory Disease
