# Supplementary material for: Relationships between prolonged physical and social isolation during the COVID-19 pandemic, reduced physical activity and disability in activities of daily living among people with advanced respiratory disease
Source: Chron Respir Dis. 2021 Aug 12;18:14799731211035822. doi: 10.1177/14799731211035822 (PMC8370888; doi:10.1177/14799731211035822)
Supplement: sj-pdf-1-crd-10.1177_14799731211035822 – Supplemental Material for Relationships between prolonged physical and social isolation during the COVID-19 pandemic, reduced physical activity and disability in activities of daily living among people with advanced respiratory disease [file sj-pdf-1-crd-10.1177_14799731211035822.pdf]

Supplementary Table 1: Differences between participants who did and did not receive a Government (GOV) letter of request to physically and socially isolate.

	Received GOV Letter	Did not receive GOV letter	Difference between groups (p value)
Age	69 [63-77]	69 [65-77]	0.77
Female	80 (46%)	11 (44%)	0.85
White British	164 (94%)	25 (100%)	0.22
Education above secondary school	77 (44%)	13 (52%)	0.47
Lives alone	58 (33%)	10 (40%)	0.66
Inpatient/residential care	4 (100%)	0	0.44
Formal caregiver	28 (16%)	1 (4%)	0.11
Informal caregiver	100 (58%)	12 (48%)	0.36
Charlson comorbidity Index score	7 [3-10]	1 [6-12]	0.24
Australian Karnofsky Performance Status	70 [60-80]	80 [60-90]	0.08
Currently physically and socially isolating	127 (73%)	15 (60%)	0.18
Have spent time in physical and social isolation	173 (99%)	20 (80%)	<0.001
Months spent in physical and social isolation	5 [3.5-8]	3.5 [0-7]	0.02
Total Barthel Index score (basic ADLs)	19 [17-20]	20 [18-20]	0.14
Lawton Brody IADL score (instrumental ADLs)	6 [5-8]	7 [6-8]	0.06
WHODAS Summary score	60 [47-81]	46.5 [40-56.5]	0.002
Symptom severity (Palliative Outcomes Scale-symptoms)	10 [6-16]	6 [4-9]	0.003
